# Cell-free synthetic biochemistry upgrading of ethanol to 1,3 butanediol

**DOI:** 10.1038/s41598-021-88899-w

**Published:** 2021-05-03

**Authors:** Hongjiang Liu, James U. Bowie

**Affiliations:** Department of Chemistry and Biochemistry, Molecular Biology Institute, UCLA-DOE Institute, University of California, 611 Charles E. Young Dr. E, Los Angeles, CA 90095-1570 USA

**Keywords:** Biocatalysis, Metabolic pathways, Biosynthesis

## Abstract

It is now possible to efficiently fix flue gas CO/CO_2_ into ethanol using acetogens, thereby making carbon negative ethanol. While the ethanol could be burned as a fuel, returning the CO_2_ to the atmosphere, it might also be possible to use the fixed carbon in more diverse chemicals, thereby keeping it fixed. Here we describe a simple synthetic biochemistry approach for converting carbon negative ethanol into the synthetic building block chemical 1,3 butanediol (1,3-BDO). The pathway completely conserves carbon from ethanol and can ultimately be powered electrochemically via formate oxidation. Our proof-of-principle system reached a maximum productivity of 0.16 g/L/h and, with replenishment of feedstock and enzymes, achieved a titer of 7.7 g/L. We identify a number of elements that can be addressed in future work to improve both cell-free and cell-based production of 1,3-BDO.

## Introduction

The chemical industry is a major producer of global warming gases, with 99% of carbon compounds derived from petroleum^1^. Utilization of captured carbon feedstock could potentially provide a 10% reduction in global carbon emissions (3.5 GT CO_2_-eq)^2^. Replacement of the carbon in organic molecules with carbon captured from CO_2_ could therefore be an important component of a truly sustainable future economy^1–3^.

Ethanol is one of the most important bio-based chemicals and is the prototype first-generation biofuel. Corn-based ethanol produced by standard yeast fermentation is currently the major source of bio-ethanol, and provides an estimated 20% reduction in greenhouse gas emissions relative to gasoline^4^. New advances enable more dramatic reductions in emissions. In particular, commercial plants are being developed that employ acetogens to fix carbon from flue gas or biomass syngas into ethanol^5,6^, potentially providing a remarkable 98% reduction in greenhouse gas emissions relative to petroleum fuel^7^. Additionally, advances in electrochemical carbon capture allow efficient conversion of CO_2_ into formaldehyde and formate^8,9^. A recent report also describes a method for efficient electrochemical conversion of CO_2_ to ethanol^10^. To the extent that the electricity used in electrochemical conversions is derived from solar or nuclear plants, electrochemistry provides another carbon negative process for making simple carbon compounds. Yet ethanol and formate currently have very limited uses due to their lack of chemical complexity and reactivity. As a result, developing effective ways to upgrade simple molecules like formate and ethanol into more diversified chemicals could potentially form the basis for a more carbon efficient chemical industry by displacing chemicals derived from petroleum^5^.

One approach to increasing product complexity of the carbon negative acetogenic process is by metabolically engineering acetogens to generate more complex chemicals^5^. Yet while a metabolic engineering approach is straightforward in theory, in practice there are many hurdles that must be overcome for commercial viability^11–13^.

Another possible approach to upgrading ethanol into more diverse products is to free ourselves from cells and employ enzyme pathways, an approach we call synthetic biochemistry^14,15^. In a pioneering effort, Zhang et al. developed a system to enzymatically convert ethanol into 2,3-butanediol and 2-butanol^16^. They first employed alcohol dehydrogenase to make acetaldehyde, which was then combined using a non-natural enzyme to make the 4-carbon molecule acetoin. Acetoin could then be reduced with dehydrogenase enzymes to make 2,3-butanediol or 2-butanol. In their system the reduction steps were powered by the oxidation of ethanol, generating NAD(P)H. Because 2,3-butanediol and 2-butanol require different reducing stoichiometries, Zhang et al. employed the purge valve concept^17^ to regulate the levels of NADPH generated in the alcohol dehydrogenase step. A limitation of powering the required reduction steps with ethanol oxidation is that the overall reaction is at the mercy of the overall thermodynamics. For example, the 2,3-butanediol conversion (2 ethanol + NAD^+^  = 2,3-butanediol + NADH) is an unfavorable reaction under standard state conditions (ΔG’^m^ = 33 kJ/mol, where ΔG’^m^ is the free energy change at 1 mM standard state and pH 7.0)^18^. Nevertheless, the purge valve system did prove effective in these examples.

Here we introduce a different approach to more flexibly deploy reducing equivalents, a way to power the reduction steps electrochemically, and we expand the range of molecules that can be made from ethanol to 1,3-BDO. In our approach, the ethanol oxidation and subsequent reduction steps are separated and we provide reducing equivalents by the oxidation of formate. As formate can be produced electrochemically from CO_2_, it introduces a way to indirectly power biochemical pathways by electricity. To make 1,3-BDO from ethanol we deploy a non-natural pathway conceived and developed by Yakunin and coworkers^19–21^.

## Results

### System design

**Figure 1:**
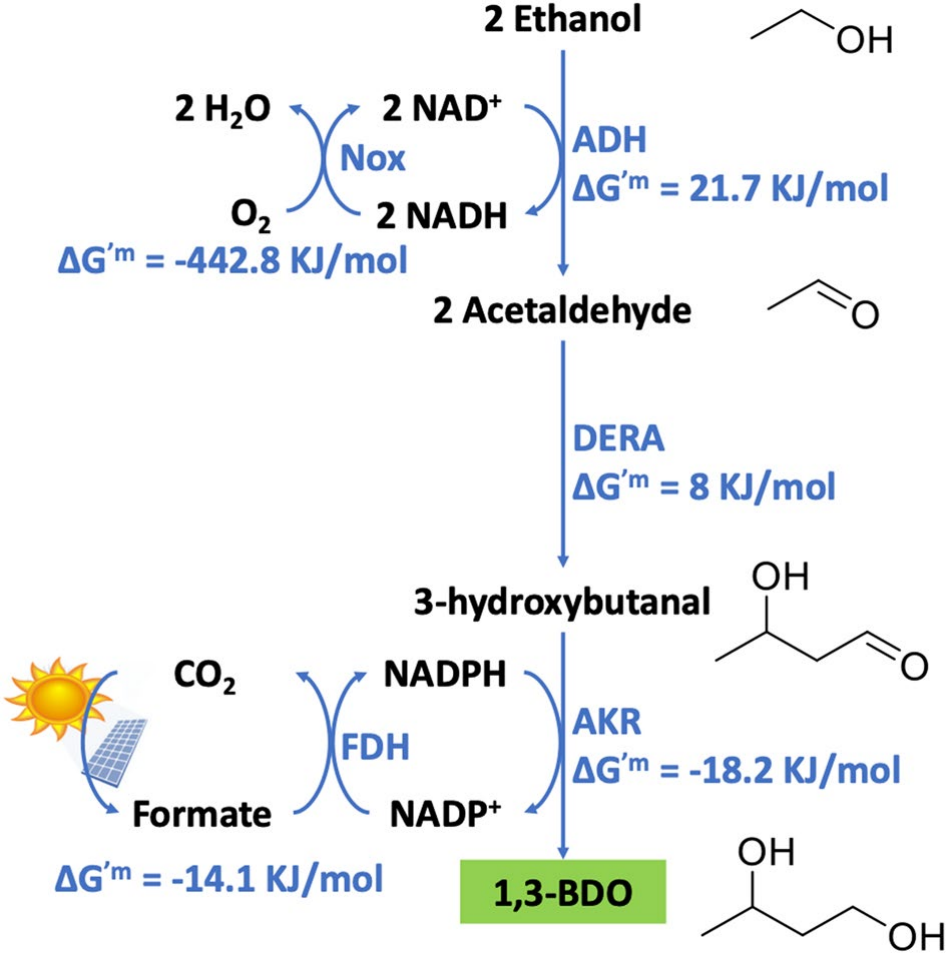
System design. Chemicals are shown in black text and enzymes in blue text. The standard state free energies for a 1 mM standard state and pH 7 are calculated using eQuilibrator^18^. As 1,3-BDO is not part of the eQuilibrator database, we substituted 2,3-BDO for the AKR reaction free energy. The chemical structures were drawn using ChemDraw v19.1 (https://www.perkinelmer.com/category/chemdraw), the solar panel image from Microsoft Powerpoint icon presets and the sun from Apple emojis.

Our system for 1,3 butanediol production from ethanol is shown in Fig. 1. Ethanol is first oxidized to acetaldehyde via alcohol dehydrogenase (ADH). Ethanol oxidation is highly unfavorable thermodynamically with a ∆G’^m^ = 21.7 kJ/mol^18^. Thus, we need a mechanism to drive the reaction forward. To accomplish this goal, we introduce an enzyme NADH Oxidase (Nox) that re-oxidizes NADH back to NAD^+^. In this manner, a large NAD^+^/NADH gradient is maintained that can drive the conversion of ethanol to acetaldehyde. To convert acetaldehyde into 1,3-BDO we used the pathway developed by Yakunin and coworkers^19–21^. To join acetaldehyde to make 3-hydroxybutanal (3-HBal) we employ a promiscuous aldolase 2-deoxy-d-ribose-5-phosphate aldolase (DERA)^22^. DERA naturally catalyzes the reversible breakdown of 2-deoxy-d-ribose-5-phosphate to glyceraldehyde 3-phosphate and acetaldehyde. DERA, however, can also catalyze an analog of the reverse reaction, to combine multiple units of acetaldehyde to hydroxyl-aldehydes such as 3-HBal^19,20,23^. Finally, 3-HBal is reduced to 1,3-BDO using a specific aldo-ketol reductase (AKR) identified by Kim et al.^21^.

To supply reducing equivalents in the form of NADPH to the biosynthetic (reductive) phase of the pathway, we employ formate dehydrogenase (FDH). In this reaction formate is converted to CO_2_. As CO_2_ can be efficiently converted back to formate electrochemically^9^, this step could ultimately be carbon neutral by using solar or nuclear power. Overall, by using carbon fixed into ethanol using acetogens and by powering the biosynthetic reactions using carbon neutral energy stored in formate, the system can provide carbon negative 1,3-BDO, and ultimately other carbon negative chemicals. The specific enzymes employed in this work are described in Supplementary Information.

### The oxidizing phase, mobilizing ethanol to acetaldehyde

**Figure 2:**
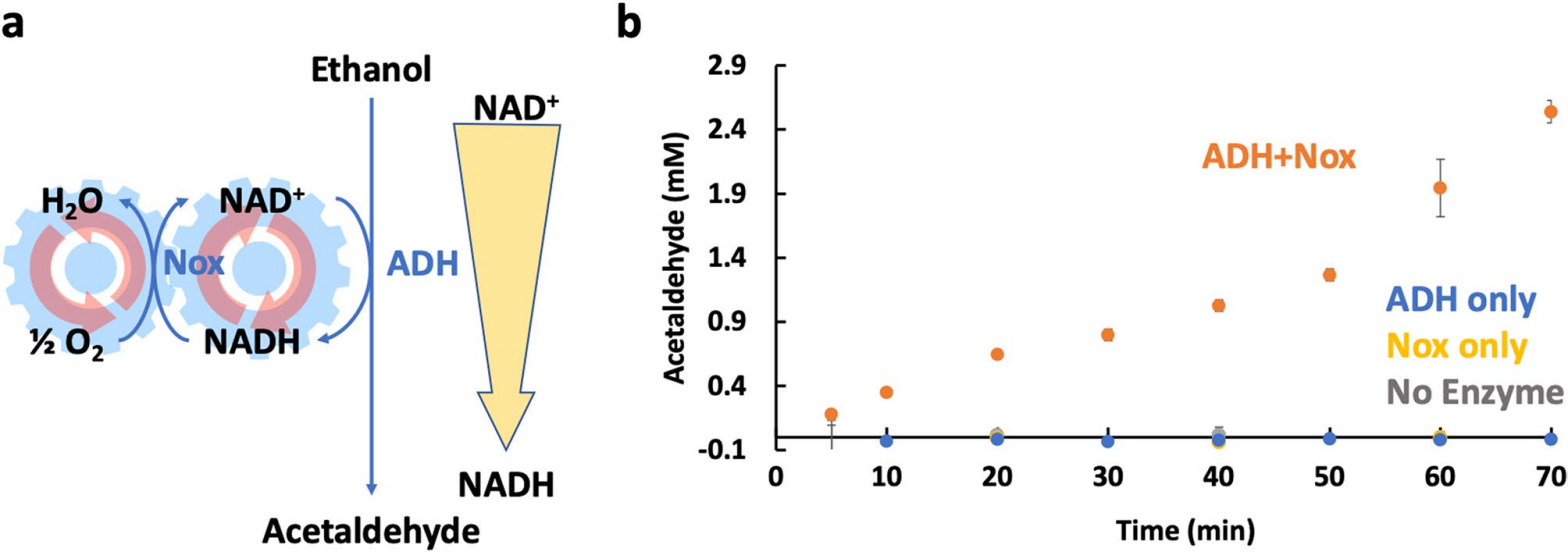
Ethanol Oxidation Module. (**a**) The reaction scheme employed emphasizing the reaction is driven by the NAD^+^/NADH gradient generated by the Nox re-oxidation of NADH to NAD^+^. (**b**) Acetaldehyde production with and without Nox. Production is improved by the introduction of Nox. Error bars reflect standard deviations of biological triplicates. All reactions were conducted with 1% ethanol and 2 mM NAD^+^ at room temperature. Concentrations of ADH and Nox are 0.011 mg/mL and 0.08 mg/mL respectively.

We first tested the effectiveness of Nox for driving the generation of acetaldehyde. As shown in Fig. 2, the addition of Nox allows for the production of acetaldehyde. Starting with 100 mM ethanol and 2 mM NAD^+^, ADH alone does not produce measurable acetaldehyde as expected given a calculated K’eq = 6.8 × 10^–4^^18^. In the presence of Nox, however, we see continual acetaldehyde production, reaching ~ 2.5 mM in 70 min. Thus, Nox is effective in facilitating acetaldehyde production as expected.

### The reductive phase: conversions of acetaldehyde to 1,3-BDO

**Figure 3:**
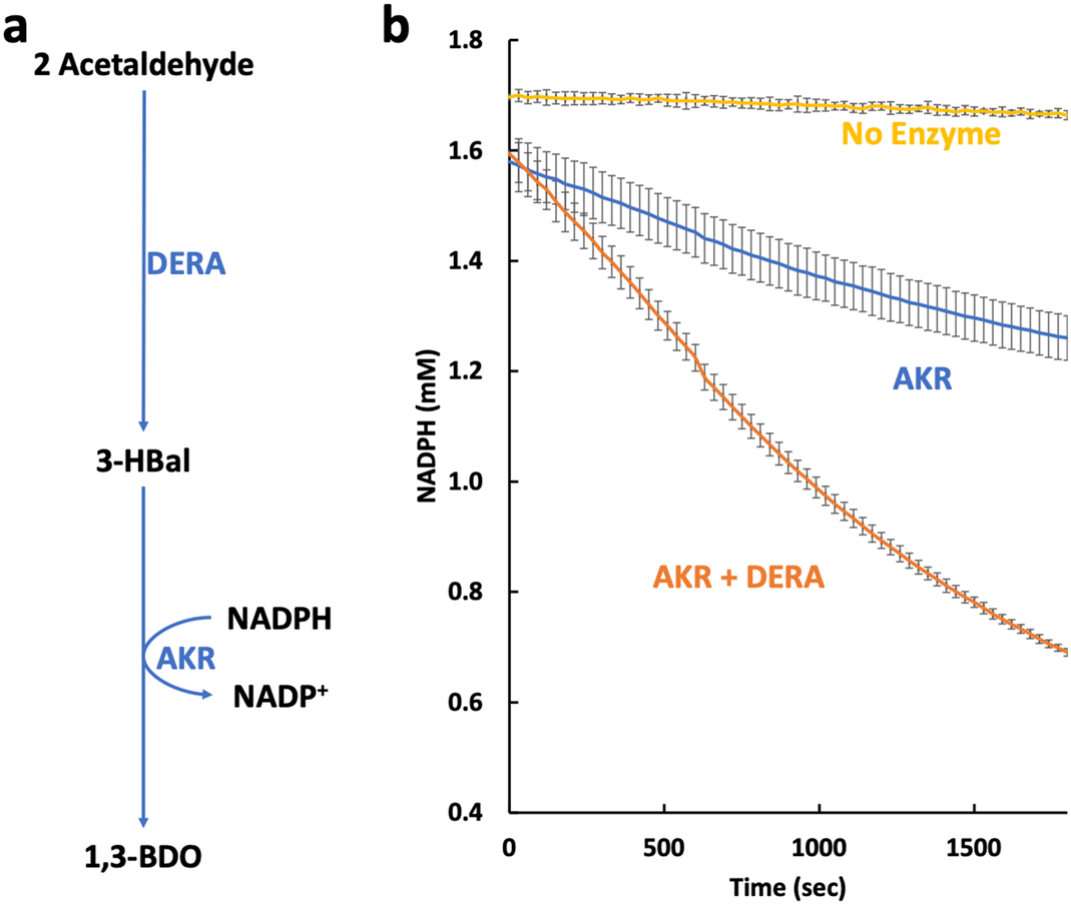
Reduction module, (**a**) schematic of the reductive module leading to 1,3-BDO. (**b**) Oxidation rates of NADPH in the presence of AKR only or AKR and DERA. The rate of oxidation is greatly enhanced in the presence of DERA, indicating the production of 3-HBal. Error bars reflect standard deviations of biological triplicates.

We next tested the biosynthetic phase of the system in which we convert acetaldehyde to 1,3 butanediol, by supplying acetaldehyde and NADPH directly. As shown in Fig. 3, when we add acetaldehyde to a mixture of DERA and AKR we see oxidation of NADPH indicative of 1,3-BDO production (direct assay for 1,3-BDO production in the full system is shown below). There is some background oxidation of NADPH with AKR alone indicating that AKR can reduce acetaldehyde, albeit at a low level. The substrate specificity of AKR is discussed further below.

### Implementation of the full pathway

With both the oxidative and the reductive modules tested, we next built the full system (Fig. 1) by putting the oxidative and reductive modules together and adding NADPH generation via formate dehydrogenase. The initial conditions based on intuitive guesses for enzyme concentrations successfully produced 1,3-BDO, albeit only 1.6 mM after 1 day. To optimize the system, we varied enzyme levels. In each optimization round we doubled and halved every enzyme independently. The highest titer enzyme loadings were then used to initiate the next round of optimization. After 7 rounds of optimizations, we saw a 25-fold titer improvement to ~ 40 mM after 1 day.

**Figure 4:**
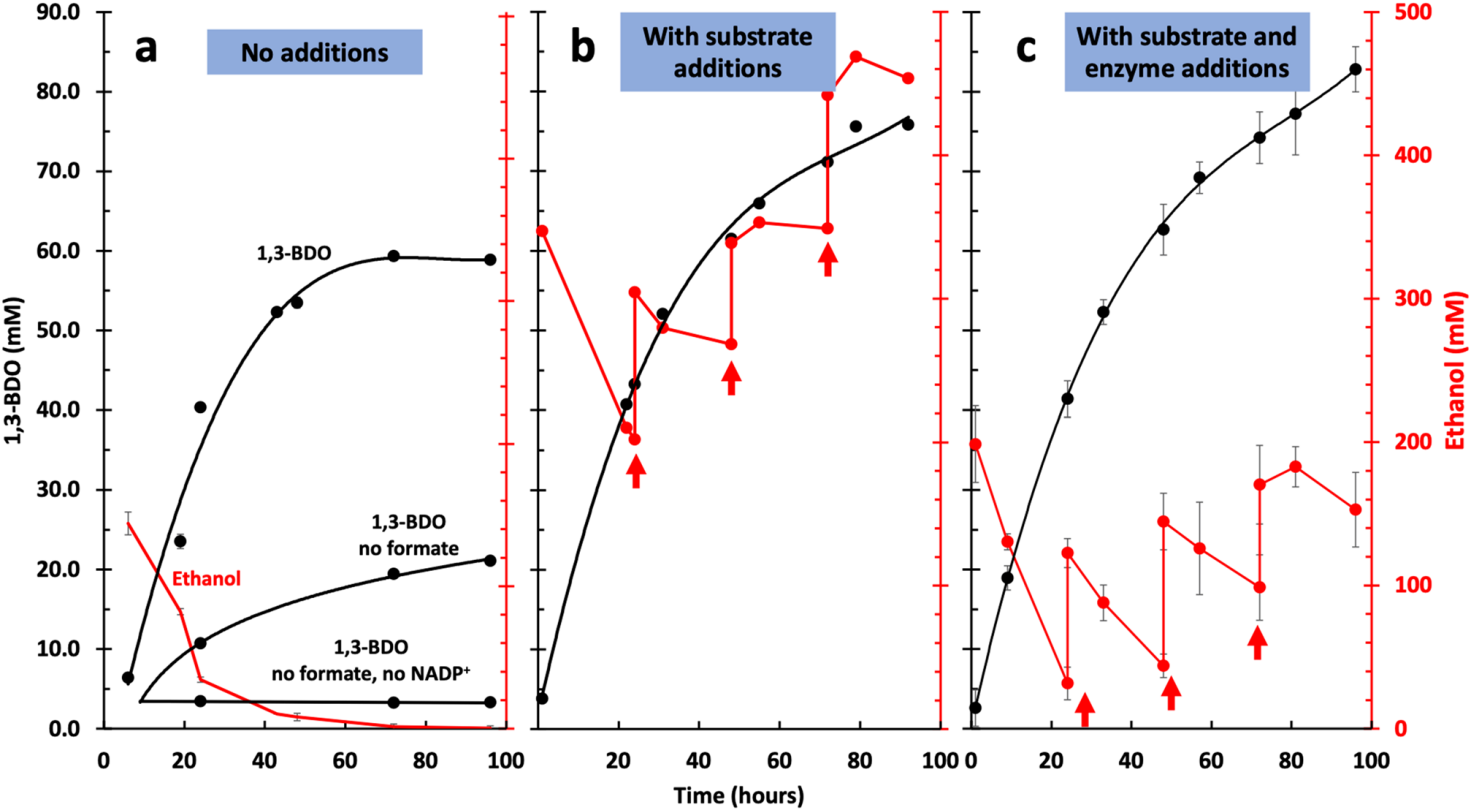
Conversion of ethanol to 1,3-BDO. Results for implementation of the full system described in Fig. 1 are shown. The 1,3-BDO concentrations are shown in black and ethanol concentrations are shown in red. (**a**) Results for the full system including results leaving out formate or leaving out formate and NADP^+^. The ethanol consumption time course corresponds to the full system. (**b**) The same implementation except that extra ethanol and formate were added at various times (indicated by the red arrows). (**c**) The same implementation except that extra formate, ethanol, Nox and DERA enzymes were added at various times (indicated by the red arrows). Error bars reflect standard deviations of biological triplicates.

A reaction time course of the optimized system is shown in Fig. 4a. Starting with 140 mM ethanol, the system produced 60 ± 0.26 mM 1,3-BDO, corresponding to about 85 ± 0.5% conversion of ethanol by day 3. All the ethanol was consumed indicating that ~ 15% of the ethanol mass accumulated in intermediates or unidentified side products.

In the absence of formate, some 1,3-BDO production occurred (Fig. 4a). We therefore suspected that there may be some cofactor cross talk. In particular, ADH may be able to utilize NADP^+^, thereby generating some NADPH that could be utilized by AKR. Alternatively, AKR could utilize NADH generated by ADH before Nox can oxidize it. To investigate these possibilities, we analyzed the cofactor specificity of ADH and AKR. The kinetic parameters are listed in Table 1. In a relevant concentration range of 1 mM cofactor, the specific activity ratio for ADH is ~ 40:1 in favor of NAD^+^ over NADP^+^ for ethanol oxidation, while AKR’s is ~ 19:1 in favor of NADP^+^ over NAD^+^ for 1,3-BDO oxidation. Thus, both enzymes are highly specific for their favored cofactors, but there is measurable crossover that could enable some production of 1,3 BDO in the absence of formate. Nevertheless, production is clearly improved with formate and if both formate and NADP^+^ were removed (Fig. 4a), we see essentially no production of 1,3-BDO indicating that the formate-NADPH regeneration system is necessary for effective 1,3-BDO production.

**Table 1:** Kinetic parameters for ADH and AKR.

Enzyme	Cofactor	K_m_ (mM)	k_cat_ (s^−1^)	k_cat_/K_m_ (s^−1^ mM^−1^)
ADH	NAD^+^	0.46 ± 0.02	1.15 ± 0.09 × 10^6^	2.5 ± 0.3 × 10^6^
	NADP^+^	≥ 8*	≥ 5.7 × 10^4^*	–
AKR	NAD^+^	6 ± 2	1.0 ± 0.2 × 10^4^	1.59 ± 0.27 × 10^3^
	NADP^+^	0.11 ± 0.03	2.84 ± 0.05 × 10^4^	2.8 ± 0.7 × 10^5^

*We were unable to sufficiently saturate ADH activity with NADP^+^ to obtain reliable kinetic parameters. We simply provide a lower bound based on the best fit to the kinetic data.

### Substrate addition to boost titer

As 140 mM ethanol was completely consumed in the optimized system, we attempted to further boost titer by raising the initial ethanol concentration to 350 mM and added new boluses of 88 mM ethanol and 30 mM formate after each day of reaction. As shown in Fig. 4b, 150 mM ethanol was consumed the first day, generating ~ 45 mM 1,3-BDO. Ethanol consumption greatly diminished the next day, however, and almost stopped after two days. While 1,3-BDO production also slowed (from ~ 1.8 to ~ 0.6 mM/h on day 2 and 3), 1,3-BDO concentrations continued to rise even in the absence of ethanol consumption, suggesting that intermediates were more slowly being converted after the initial ethanol consumption.

### Enzyme inactivation

**Figure 5:**
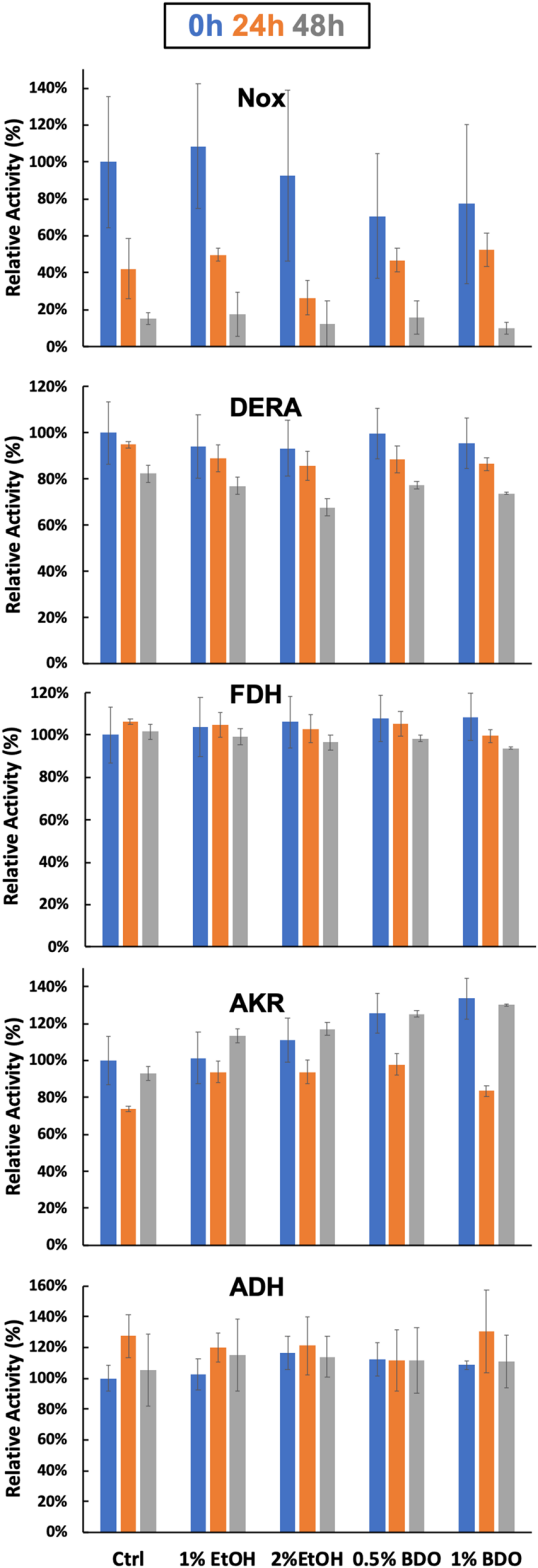
Enzyme inactivation. The activity of the enzymes after 0 (blue bars), 24 (orange bars) and 48 (grey bar) hours of incubation in the absence (Ctrl) or presence of various alcohols as indicated in the horizontal axis. Relative activity refers to the activity as a percentage of the control at 0 h. Error bars reflect standard deviations of biological triplicates.

We next sought to learn why the reactions slow down and then stop after several days. It is possible that the enzymes become inactivated by ethanol or the increasing 1,3-BDO concentrations. We therefore tested the inactivation of each of the enzymes when challenged with ethanol or 1,3-BDO. As shown in Fig. 5, ADH, FDH and AKR are all stable for several days in both ethanol and 1,3-BDO in the relevant concentration ranges. DERA is also relatively stable but loses about 20% activity after 2 days in the absence of solvents. DERA’s activity loss is only modestly accelerated by the alcohols, losing ~ 35% of its activity in 2% ethanol and ~ 25% of its activity in 1% BDO. Nox, however, loses ~ 90% of its activity after 2 days regardless of solvent conditions. Nox inactivation could therefore explain the slowing and ultimate cessation of ethanol consumption after several days due to buildup of NADH, while DERA inactivation is a possible cause of the slow down of 1,3-BDO production at later times.

### Enzyme replenishment

In an effort to prolong the reactions, we tested additions of fresh Nox and DERA enzymes that are susceptible to inactivation. We repeated the substrate addition experiments while also adding fresh enzyme additions. Starting with 200 mM ethanol, we added 88 mM ethanol and 30 mM formate every 24 h as before, but also added Nox and DERA enzymes in proportion to their expected loss of activity. With this approach the reactions continued producing 1,3-BDO for 4 days, generating a titer of 85 mM or 7.7 ± 0.25 g/L (Fig. 4c) with an initial productivity over the first day 0.16 ± 0.006 g/L/h. The final yield was 85% of theoretical in the single feed reaction and 54% in the multiday replenishment reaction.

### Enzyme specificity and product chirality

Although the *P. aeruginosa* AKR employed here was previously identified from an extensive screen as highly active and specific for 3-HBal^21^, 3-HBal is a chiral molecule and the stereochemical preference of AKR has not been established. Moreover, while AKR was reported to have negligible activity with acetaldehyde^21^, we noted some ability to reduce acetaldehyde (Fig. 3b). So, we decided to investigate AKR specificity further. We were unable to obtain stereochemically pure 3-HBal for this investigation, but we were able were able to purchase stereochemically pure (R) 1,3-BDO, along with the racemic mixture. We therefore decided to investigate stereochemical specificity using the oxidation reaction from 1,3-BDO to 3-HBal.

**Figure 6:**
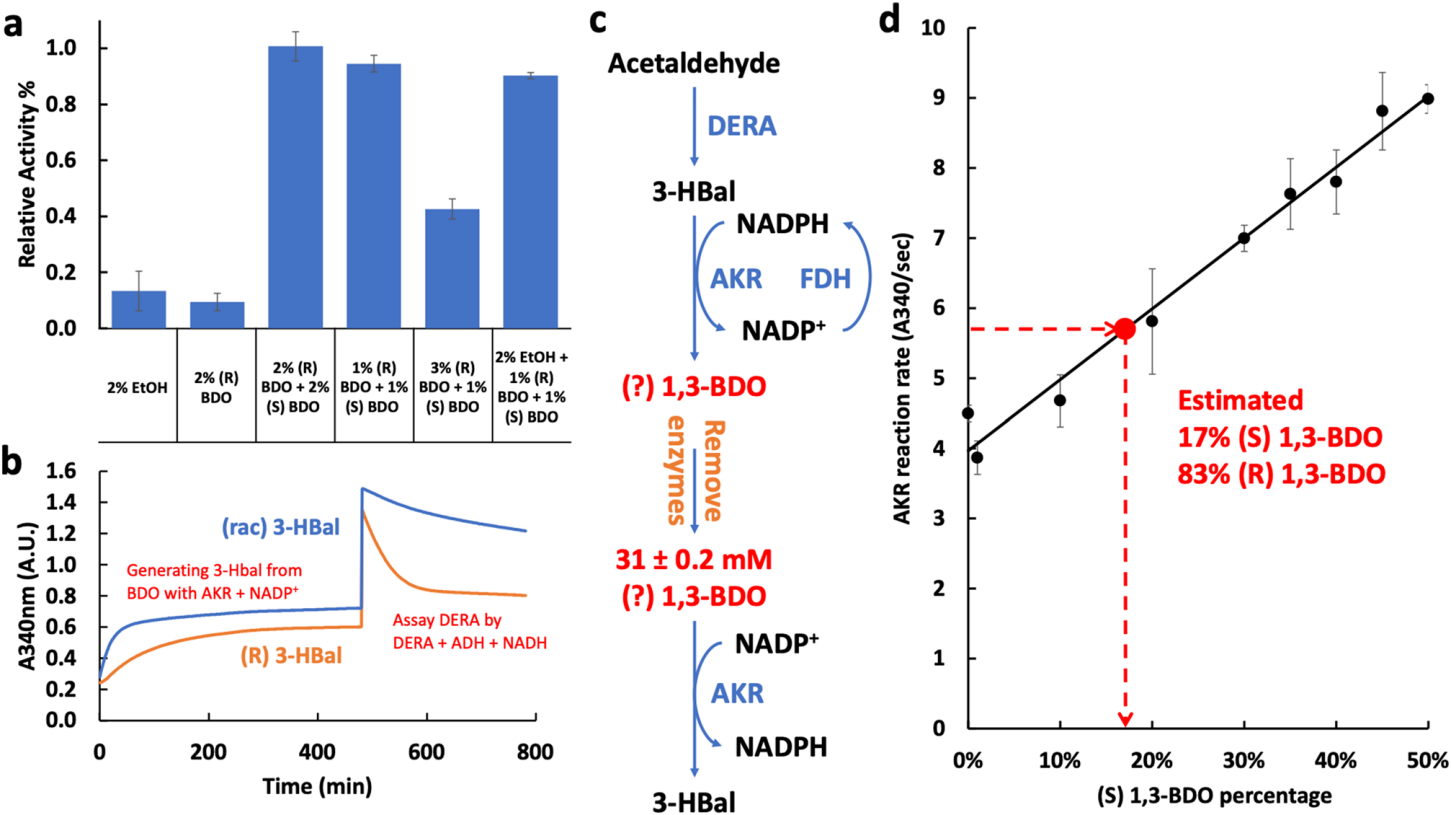
Stereochemical preference of enzymes and product composition. (**a**) Relative AKR reaction rates for oxidation of various alcohols. AKR has a strong preference for (S) 1,3-BDO. Relative activity is the fractional rate compared to 2% (R) + 2% (S) 1,3-BDO. (**b**) DERA reaction rates using equal concentrations of the substrates (R) or (R,S) 3-HBal. We assessed DERA specificity in two phases. In the first phase, we generated (R) or (R,S) 3-HBal from 1,3-BDO using AKR and NADP^+^. In the second phase, we then measured the activity of DERA on the two different stereoisomeric populations of 3-HBal by the addition of DERA, ADH and NADH. In this second phase, DERA generates acetaldehyde, which is detected by ADH reduction to ethanol and concomitant oxidation of ethanol. The reduction of NADP+ in the first phase and the oxidation of NADH in the second phase can both be monitored by absorbance at 340 nm. The results indicate that (R) 3-HBal is the preferred substrate. (**c**) Scheme for assessing the stereochemical composition of the 1,3-BDO product of the DERA/AKR system. First, the 1,3-BDO product of unknown stereochemical composition was made from acetaldehyde using DERA and AKR enzymes as in the full enzyme system. Then the enzymes were removed and the concentration of the 1,3-BDO was determined (31 mM). To assess the stereochemical composition, we then measured the rate of the AKR catalyzed reaction back to 3-HBal. This rate was compared to the rates of the same AKR catalyzed reaction using substrates of known stereochemical composition (**d**). (**d**) The standard curve measuring the rate of the AKR catalyzed conversion of 31 mM 1,3-BDO to 3-HBal at different stereochemical compositions. Comparing to the known reaction rates to the rate obtained for the product of converting acetaldehyde to 1,3-BDO using DERA and AKR (red dot), allows us to estimate the stereochemical composition of the product at ~ 17% (S) 1,3 BDO. Error bars reflect standard deviations of biological triplicates.

As shown in Fig. 6a, AKR shows similar activity with ethanol and (R) 1,3-BDO, but is about tenfold more active with the racemic mixture. This result indicates that AKR is highly specific for (S) 1,3-BDO. Indeed, (R) 1,3-BDO is somewhat inhibitory (compare rates with 1% (S) 1,3-BDO in the presence of 3% and 1% (R)). Our finding that AKR is specific for (S) 1,3-BDO was a surprise because based on the natural reaction, DERA is expected to make (R) 3-HBal and the same AKR used here can make (R) 1,3-BDO in vivo^20^. If so, it implies that 1,3-BDO production occurs in spite of a stereochemical mismatch.

To investigate this possibility further, we sought to validate the assumed stereochemical preference of the DERA enzyme for making (R) 3-HBal. As we could not purchase stereoisomers of 3-HBal, we first used AKR to oxidize (R) or (R,S) 1,3-BDO to 3-HBal, thereby generating either (R) 3-HBal or (R,S) 3-HBal (Fig. 6b). We then assessed DERA activity on the generated substrates. As shown in Fig. 6b, the DERA enzyme is much more active with the (R) substrate than with the mixed substrate, confirming that DERA prefers the (R) stereoisomer. Thus, there is an unexpected stereochemical mismatch between DERA and AKR.

Our results indicate that the DERA enzyme preferentially makes the (R) stereoisomer, but AKR prefers (S). Moreover, AKR has similar activity with (R) 1,3-BDO and ethanol. But this presents a conundrum because the results shown in Fig. 3b reveals that AKR is more reactive with the product of the DERA reaction than with acetaldehyde. To explain this apparent dichotomy, we suspected that DERA must produce at least some (S) 3-HBal along with (R) 3-HBal. If so, then the product would also contain some (S) 1,3-BDO. To test this possibility, we used DERA and AKR to generate 1,3-BDO from acetaldehyde. We then evaluated the chirality of the product by measuring the reaction rate of reverse reaction from 1,3-BDO to 3-HBal and compared it to the rates using various known ratios of (R) and (S) 1,3-BDO (see scheme in Fig. 6c). As shown in Fig. 6d, the reaction rate for the 1,3-BDO product suggests that the product indeed contains 17 ± 2.5% (S) 1,3-BDO.

## Discussion

In this work we have taken steps to test and expand ability of cell-free platforms to upgrade ethanol. Our approach adds flexibility by separating the oxidation of ethanol from the reduction step required for 1,3-BDO biosynthesis. The production parameters achieved here (0.16 g/L/h productivity, 7.7 g/L titer and 75–85% yield) are higher than achieved in cells so far (1.1 g/L titer, < 5% yield from glucose)^20^, but will clearly require considerable improvement for commercial viability. Nevertheless, there are obvious avenues for improvement through genome mining or enzyme engineering. In particular, we discovered a chirality mismatch between the DERA and AKR enzymes that have been previously developed to make 1,3-BDO in vivo. The previously identified AKR enzyme prefers (S) 3-HBal, but DERA predominantly makes (R) 3-HBal. Apparently DERA makes a small amount of (S) 3-HBal and, coupled with the preference of AKR for (S) 3-HBal, the final product is a mixture of (R) and (S) 1,3-BDO. It should be possible to improve the rate of the reaction dramatically if the correct chiral substrate could be delivered to AKR or a different AKR specific for (R) 3-HBal could be identified. Repairing this mismatch could also be a boon for cell-based production of 1,3-BDO. Another notable issue is the poor stability of the Nox enzyme in the presence of alcohols. Engineering or finding a more stable variant could address this problem, however^15^. An advantage of the cell-free approach is that these issues can be readily identified, making avenues for improvements clear. Ultimately, we hope that continued efforts to upgrade ethanol using enzyme pathways could provide a path to diversifying current ethanol markets, and ultimately lead to a panoply of carbon negative chemicals in which the carbon is sourced from the atmosphere rather than extraction from the ground.

## Methods

### Molecular cloning

All genes are codon optimized for *E. coli* expression (for DNA sequences please see Supplementary Information). The genes were either synthesized and cloned into the Nde1-Xho1 site of a pET28b plasmid or cloned in-house into the same site on pET28b and the sequences verified. Gene synthesis was performed by Twist Bioscience and gene sequencing by Genewiz. For expression, all plasmids were transformed into *E. coli* Bl21 (DE3) Gold except for FDH which was transformed into *E. coli* C43.

### Protein expression and purification

Expression of all enzymes except FDH was performed by growing in 1 L of LB auto-induction media with 50 µg/mL kanamycin at 37 °C for 20 h. Auto-induction media was prepared by adding 0.5 g glucose and 2 g lactose mixture into Miller’s formula LB medium. FDH was expressed by growing cells at 37 °C to OD600 ~ 0.8 in LB medium with 50 µg/mL kanamycin, induction with 1 mM IPTG and incubation overnight at 18 °C. Cultures were centrifuged at 3720×*g* for 30 min and the cell pellets were resuspended and incubated in 20 mL Hypotonic Lysis Buffer (50 mM NaCl, 20 mM Tris–HCl, pH 7.5) with 2.5 mg chicken egg-white lysozyme (Sigma-Aldrich) for 5 min. The cells were lysed using an Avestin Emulsiflex C3 homogenizer at 10,000 bars. Then 2500 units of benzonase nuclease (Sigma-Aldrich) were added to the lysate and centrifuged at 24,500×*g* for 30 min. The clear supernatant was incubated with 2.5 mL (packed volume) of Ni–NTA for 30 min at 4 °C. The mixture was then applied to a gravity column. The beads were washed three times with 7.5 mL of Hypotonic Lysis Buffer and then two times with 7.5 mL bed volume of Wash Buffer (150 mM NaCl, 20 mM Tris–HCl, pH 7.5, 10 mM imidazole). The enzymes were eluted with Elution Buffer (150 mM NaCl, 50 mM Tris–HCl pH 7.5, 250 mM imidazole, 10% glycerol). Protein concentration was determined by absorbance at 280 nm using calculated extinction coefficients predicted from the primary sequence by the EXPASY server. Nox enzymes solutions were brilliantly yellow. For long term storage, all enzymes were flash-frozen in Elution Buffer using liquid nitrogen and stored in − 80 °C.

### Ethanol oxidation module

Reactions were performed in 2 mM NAD^+^ and 100 mM ethanol in the General Buffer (10 mM KCl, 50 mM NaCl, 10 mM MgCl2 and 100 mM Tris–HCl, pH 7.5). 100 µL reactions were initiated by adding ADH to a final concentration of 0.01 mg/mL, or a mixture of ADH or Nox at final concentrations of 0.01 mg/mL and 0.08 mg/mL, respectively. The reaction was monitored by absorbance at 340 nm, recorded in a SpectraMax M35 plate reader. Acetaldehyde was directly assayed using a purpald assay. 10 μL of the reaction mixtures were added to 90 μL of purpald reagent (5 g/L purpald in 0.5 M NaOH). After incubation at room temperature for 10 min, the absorbance at 550 nm was measured. Acetaldehyde concentrations were determined by comparison to a standard curve developed using known concentrations of acetaldehyde.

### Reductive module and substrate specificity

The reactions consisted of 50 mM acetaldehyde and 2 mM NADPH in the General Buffer. Reactions were initiated by the addition of 0.1 mg/mL (final) AKR with or without 1.5 mg/mL (final) DERA and the reactions monitored by absorbance at 340 nm.

### 1,3-BDO production

Optimized single batch BDO production consisted of the following mixture in General Buffer: 1 mM of NADP^+^, 4 mM of NAD^+^, 140 mM ethanol, 50 mM formate (pH = 7.4) and enzymes (0.13 g/L of ADH. 0.51 g/L of Nox, 3 g/L of DERA, 0.28 g/L of AKR and 0.29 g/L of FDH). 200 µL solutions were placed in 12 mL threaded glass tubes and the tubes were sealed tight and incubated at 29 °C with mixing on a rotating drum. The reactions were stopped at various timepoints by freezing at − 80 °C. After the vessels were brought to room temperature together, the reactions were extracted with 200 μL 1-hexanol by vigorous mixing followed by centrifugation. The organic phase was collected for analysis by GC-FID (Thermo Scientific TRACE 1310 GC) or GC–MS (Agilent 6890 Gas Chromatograph, and 5975 Inert Mass Selective Detector) as described below. The titer was determined by comparison to a calibration curve of analytical 1,3-BDO prepared in similar manner.

The optimized reactions with replenishment were initiated in the same manner as the optimized single batch BDO production as described above. Every 24 h the reactions were replenished by the addition of fresh air, 88 mM ethanol, 50 mM formate pH 7.4, 0.27 g/L Nox and 0.75 g/L DERA (total replenishment volume of 13 μL). Then the tubes were resealed and incubation continued. Reactions were processed as described above.

### Gas-chromatography analysis

For GC-FID, 1 μL samples from the organic phases were injected into a Thermo Scientific TRACE 1310 GC system with FID with a Thermo-Scientific TG-WAXMS column with dimensions of 30 m × 320 μm × 0.25 μm. Helium was used as carrier with the flow set to 30 mL min^−1^ in constant flow mode. The FID was ignited with 350 mL min^−1^ ultrapure air and 35 mL min^−1^ hydrogen at constant flow. Data was analyzed with Chromeleon 7 software. For BDO detection the initial oven temperature was set to 50 °C for 2 min followed by a 10 °C min^−1^ ramp to 140 °C, then a second temperature ramp of 50 ℃ min^−1^ to a final temperature of 235 °C, which was maintained for 3 min. Both inlet and detector temperatures were set at 250 °C.

### Stereochemical assessment assays

AKR assays employed 0.18 mg/mL AKR and 2 mM NADP^+^ in General Buffer. Reactions were initiated by the addition of ethanol, R-1,3-butanediol or rac-1,3-butanediol and the reactions monitored by absorbance at 340 nm to obtain initial rates.

To assess DERA stereospecificity we first generated (R) or (R/S) 3-HBal from 1,3-BDO using AKR. We oxidized 1% 1,3-BDO to 3-HBal in General Buffer using 0.18 mg/mL AKR and 1 mM NADP^+^ for about 8 h. We then assessed DERA activity on the 3-HBal product by adding 1.6 mg/mL DERA and 0.21 mg/mL ADH and 1 mM NADH. In this reaction, DERA catalyzes the conversion of the 3-HBal product to acetaldehyde, which is then conveniently monitored by conversion to ethanol.

To assay the stereochemical composition of the 1,3-BDO produced by the combination of DERA and AKR, we produced 1,3-BDO using 100 mM acetaldehyde as feedstock with 50 mM formate and 1 mM NADP^+^ to drive the reductive phase for 24 h. The enzyme loading was same as in the optimized system. After the reaction was complete, enzymes were removed by passage through a 3 KDa cut-off membrane (Amicon Ultra-15 3KDa). The flow-through was collected and 1,3-BDO concentration measured to be 31 mM using the GC-FID as described above. We then measured the rate of AKR oxidation of the product back to 3-HBal by adding 0.18 mg/mL AKR and 5 mM NADP+, monitoring NADPH production by absorbance at 340 nm. The rate was compared to the rate obtained for a series of standards with known stereochemical mixtures.at the same 1,3-BDO concentration as the product (31 mM).

### ADH and AKR kinetic parameters

Michaelis–Menten kinetic parameters were obtained using 1% ethanol for ADH or 1% (R,S) 1,3-BDO for AKR. We used 0 to 2.5 mM NAD^+^ or NADP^+^ for ADH and 0 to 5 mM cofactors for AKR. Initial rates of reactions were recorded at room temperature in General Buffer by measuring absorbance at 340 nm spectroscopy. Rate-concentration curves were fit to a Michaelis–Menten kinetic model to obtain kinetic parameters.

## Supplementary Information

Supplementary Information.
