# Supplementary material for: Cell-free synthetic biochemistry upgrading of ethanol to 1,3 butanediol
Source: Sci Rep. 2021 May 3;11:9449. doi: 10.1038/s41598-021-88899-w (PMC8093283; doi:10.1038/s41598-021-88899-w)
Supplement: Supplementary file 1 — Supplementary Information. [file 41598_2021_88899_MOESM1_ESM.docx]

# **Supplementary Information for**

Cell-free synthetic biochemistry upgrading of ethanol to 1,3 butanediol

Hongjiang Liu and James U. Bowie*

Department of Chemistry and Biochemistry, Molecular Biology Institute, UCLA-DOE Institute, University of California, Los Angeles, CA

**Supplementary Table 1. The enzymes used**

| **Enzyme** | **E.C.** | **Uniprot ref.** | **Source** |
| --- | --- | --- | --- |
| ADH | 1.1.1.1 | P12311 | *Geobacillus stearothermophilus* |
| Nox | 7.1.1.2 | See note * | *Lactobacillus brevis* |
| FDH | 1.17.1.9 | B5A8W5 | *Burkholderia stabilis* |
| DERA | 4.1.2.4 | Q9X1P5 | *Thermotoga maritima* |
| AKR | 1.1.1.21 | A0A072ZLB8 | *Pseudomonas aeruginosa* |

The stability of the enzyme was improved using the PROSS algorithm: Goldenzweig, A. et al. Automated Structure- and Sequence-Based Design of Proteins for High Bacterial Expression and Stability. Mol. Cell 63, 337–346 (2016).

**DNA sequences encoding for the enzymes used in this work**

**ADH:**

ATGAAAGCTGCAGTTGTGGAACAATTTAAAAAGCCGTTACAAGTGAAAGAAGTGGAAAAACCTAAGATCTCATACGGGGAAGTATTAGTGCGCATCAAAGCGTGTGGGGTATGCCATACAGACTTGCATGCCGCACATGGCGACTGGCCTGTAAAGCCTAAACTGCCTCTCATTCCTGGCCATGAAGGCGTCGGTGTAATTGAAGAAGTAGGTCCTGGGGTAACACATTTAAAAGTTGGAGATCGCGTAGGTATCCCTTGGCTTTATTCGGCGTGCGGTCATTGTGACTATTGCTTAAGCGGACAAGAAACATTATGCGAACGTCAACAAAACGCTGGCTATTCCGTCGATGGTGGTTATGCTGAATATTGCCGTGCTGCAGCCGATTATGTCGTAAAAATTCCTGATAACTTATCGTTTGAAGAAGCCGCTCCAATCTTTTGCGCTGGTGTAACAACATATAAAGCGCTCAAAGTAACAGGCGCAAAACCAGGTGAATGGGTAGCCATTTACGGTATCGGCGGGCTTGGACATGTCGCAGTCCAATACGCAAAGGCGATGGGGTTAAACGTCGTTGCTGTCGATTTAGGTGATGAAAAACTTGAGCTTGCTAAACAACTTGGTGCAGATCTTGTCGTCAATCCGAAACATGATGATGCAGCACAATGGATAAAAGAAAAAGTGGGCGGTGTGCATGCGACTGTCGTCACAGCTGTTTCAAAAGCCGCGTTCGAATCAGCCTACAAATCCATTCGTCGCGGTGGTGCTTGCGTACTCGTCGGATTACCGCCGGAAGAAATACCTATTCCAATTTTCGATACAGTATTAAATGGAGTAAAAATTATTGGTTCTATCGTTGGTACGCGCAAAGACTTACAAGAGGCACTTCAATTTGCAGCAGAAGGAAAAGTAAAAACAATTGTCGAAGTGCAACCGCTTGAAAACATTAACGACGTATTCGATCGTATGTTAAAAGGGCAAATTAACGGCCGCGTCGTGTTAAAAGTAGATTAA

**Nox PROSS mutant:**

ATGAAAGTTACAGTGGTTGGGTGTACGCACGCCGGCACGTTTGCAATTAAGCAAATTCTGAAAGAGCACCCAGACGCAGAAGTCACCGTCTACGAACGTAACGATGTGATCTCATTTCTGTCGTGCGGAATCGCCCTGTATTTAGGAGGGCAGGTGAAGGACCCACAAGGATTGTTTTATTCCTCGCCAGAGGAATTACAGAAACTTGGTGCCAATGTCCAAATGAATCATAATGTTTTGGCCATCGATCCGGATAACAAAACAGTTACCGTCGAAGATTTGACGAATGGGGAGCAGTTTACTGAAAGTTATGACAAGTTGGTAATGACATCTGGATCGTGGCCGATCGTGCCTAAAATCCCCGGTATCGACTCCGACCGCGTGCAGCTGTGTAAAAATTGGGCGCATGCCCAAGAGCTTTACGAGCGCGCAAAAGAGGCGAAGCGTATTGTCGTTATTGGAGCGGGCTATATCGGTGCAGAATTGGCCGAGGCTTATAGCACAACGGGGCATGATGTAACTCTGATTGATGCGATGGCGCGTGTCATGCCGAAATATTTCGACAAAGAATTCACTGACGTGATCGAACAGGACTACCGCGACCATGGTGTCCAGTTAGCACTTGGAGAGACAGTGGAATCGTTCGAGGATTCAGCCAATGGTCTGACTATTAAAACTGACAAGGGGTCTTATGAGACTGATTTAGCAATTCTTTGTATCGGGTTTCGCCCAAACACTGATTTATTAAAGGGCAAGGTGGATATGTTACCAAATGGTGCCATCATCACCGACGATTATATGCGCTCCTCCAACCCGGATATTTTCGCCGCTGGCGATTCTGCCGCCGTTCATTACAACCCTACTCATCAATATACTTACATCCCTCTTGCTACGAACGCTGTACGCCAGGGTATCCTGGTTGGGAAAAATTTGGTTAAGCCAACCGTGAAGTACATGGGCACACAGTCCAGCTCTGGATTAGCTCTGTACGATCGCACCATTGTAAGCACCGGCTTAACGCTGGAGGCCGCAAAACAACTTGGCTTAAACGCCGCGCAGGTGATTGTAGAAGATAATTACCGTCCTGAGTTTATGCCGACCACGGAGCCGGTGTTAATGTCCCTTGTGTACGATCCCGACACTCATCGCATCTTGGGTGGTCAGCTGATGTCTAAGTATGATGTCAGTCAGTCCGCCAACACTTTGTCGGTTTGCATCCAGAACAAAATGACAATTGACGACCTGGCCATGGTCGACATGCTTTTTCAGCCCAACTTTGATCGCCCTTGGAACTACCTTAATATTCTGGCGCAGGCTGCTCAAGCGAAGGTGGCGCAATCGGTAAACTAA

**FDH:**

ATGGGCCTGGTGCCGCGCGGCAGCCATATGGCCACTGTATTATGCGTTCTTTATCCCGACCCCGTTGATGGGTATCCGCCGCATTATGTCCGTGATACTATCCCCGTTATCACTCGTTATGCTGACGGCCAAACGGCCCCGACACCTGCTGGTCCCCCGGGCTTTCGTCCTGGGGAATTAGTGGGATCAGTCTCGGGCGCTCTTGGTCTTCGCGGCTATCTTGAGGCGCATGGTCACACTCTTATTGTAACGTCAGATAAAGATGGGCCGGACTCCGAATTTGAGCGCCGCTTGCCAGATGCAGATGTTGTCATTAGCCAGCCGTTTTGGCCTGCGTATTTGACGGCAGAGCGTATTGCGCGCGCCCCCAAACTTCGTCTTGCGCTGACTGCAGGTATCGGTAGCGACCACGTTGATTTAGACGCGGCGGCTCGTGCTCATATCACAGTGGCAGAGGTTACTGGGAGCAACTCGATCTCTGTTGCCGAACACGTTGTAATGACCACGTTGGCTTTAGTCCGTAATTATTTACCGTCACATGCGATTGCCCAACAGGGCGGCTGGAACATTGCCGACTGTGTTTCCCGCAGCTACGATGTCGAAGGTATGCATTTTGGTACAGTTGGGGCTGGGCGCATCGGATTGGCGGTACTGCGTCGCCTTAAGCCATTTGGTCTGCACTTACACTATACCCAACGTCACCGTTTGGACGCCGCCATCGAACAAGAATTAGGTCTGACCTATCACGCTGATCCCGCGTCGCTTGCAGCAGCTGTAGATATCGTTAATTTGCAAATTCCACTTTACCCCTCAACTGAGCACCTTTTTGACGCGGCGATGATTGCCCGTATGAAGCGCGGAGCCTACTTAATTAATACGGCGCGCGCTAAATTAGTCGATCGTGATGCAGTAGTTCGTGCGGTTACGTCAGGCCATCTTGCTGGTTATGGGGGTGACGTATGGTTTCCGCAGCCCGCACCGGCAGACCATCCGTGGCGTGCTATGCCGTTCAATGGGATGACACCTCATATTTCAGGAACAAGTCTTTCTGCTCAAGCACGTTACGCGGCTGGTACCCTTGAGATCTTGCAGTGCTGGTTCGACGGTCGCCCTATTCGTAATGAGTACCTGATCGTAGATGGAGGTACACTTGCGGGTACGGGGGCGCAGAGCTATCGTCTTACCTAA

**DERA:**

ATGATCGAGTACCGCATTGAGGAAGCCGTCGCCAAGTATCGTGAGTTTTATGAGTTTAAGCCTGTGCGCGAGAGCGCCGGCATTGAGGACGTTAAGTCAGCAATTGAGCATACCAACCTCAAGCCATTTGCCACACCTGATGATATCAAGAAGTTATGTCTGGAAGCGCGTGAAAATCGCTTTCACGGCGTGTGCGTTAATCCATGTTATGTTAAGCTTGCGCGCGAAGAATTAGAGGGCACCGACGTGAAGGTCGTCACAGTGGTTGGCTTCCCCCTCGGGGCGAACGAGACCCGCACTAAGGCACACGAGGCTATCTTCGCGGTAGAAAGTGGCGCCGACGAGATTGACATGGTCATCAATGTTGGCATGCTGAAAGCGAAGGAATGGGAATACGTGTATGAGGATATCCGTTCGGTGGTGGAGAGCGTCAAGGGTAAGGTGGTAAAAGTGATTATCGAGACCTGTTATTTAGATACTGAGGAGAAGATCGCGGCATGCGTGATCAGCAAGCTGGCGGGCGCTCATTTCGTGAAGACTTCGACGGGATTTGGTACCGGTGGTGCGACAGCGGAAGACGTGCATTTGATGAAGTGGATCGTGGGAGATGAGATGGGTGTGAAGGCGTCCGGTGGTATTCGTACCTTCGAAGATGCTGTCAAGATGATTATGTATGGCGCAGACCGCATTGGCACATCAAGTGGCGTAAAGATTGTACAAGGCGGTGAGGAGCGTTACGGTGGTTGA

**AKR:**

ATGTCAGTGGAAAGCATCCGCATTGAAGGCATCGACACCCCAGTTAGTCGTATTGGGTTGGGTACCTGGGCGATTGGTGGATGGATGTGGGGCGGCGCGGACGACGCGACCAGCGTTGAGACGATCCGTCGTGCTGTGGAAAGTGGTATTAATCTCATCGATACAGCACCCGTGTATGGATTCGGCCACTCAGAAGAGGTCGTCGGAAAGGCCCTGCAAGGTTTGCGCGATAAGGCGGTGATTGCAACGAAAGCAGCGCTTGAGTGGAGCGATGCAGGTATTCACCGCAATGCTTCGGCCGCCCGTATCCGTCGCGAGGTTGAGGACTCTCTGCGCCGCTTGAAGACTGATCGTATCGACCTCTACCAGATTCATTGGCCGGACCCTCTTGTGGCTCATGAAGAGACAGCTGGTGAGCTCGAGCGCCTGCGTCGCGACGGCAAGATTCTCGCAATTGGCGTGAGCAATTATTCACCAGAGCAGATGGACGGGTTCCGCCAATTTGCACCATTGGCATCGGTCCAACCACCCTACAATCTTTTCGAGCGTGCAATCGACGCGGACGTCCTTCCCTATGCAGAACGCAACGGTATCGTTGTATTGGCATATGGCGCGCTTTGCCGCGGTTTGTTATCTGGCCGCATGAATGCGGAGACCCGCTTCGACGGTGATGACCTGCGTAAGAGCGACCCAAAATTTCAACAACCTCGCTTCGCGCAATATCTGGCGGCAGTAGCGCAGTTAGAGGAGTTAGCACGTGAGCGTTATGGAAAGAGTGTATTAGCTCTCGCAATCCGCTGGATTCTCGATCGTGGCCCGACAGTGGCACTGTGGGGAGCGCGCAAACCAGAGCAATTAAATGGAATCGCGGACGCATTTGGGTGGCGTCTCGACGACGAGGCAATGGCGCGCATCGAACGCATCTTAGCGGAGACCATTCAGGACCCGGTAGGCCCCGAGTTTATGGCGCCGCCAAGTCGTAATGCGTGA
